# Serologic Responses to Recombinant *Pneumocystis jirovecii* Major Surface Glycoprotein among Ugandan Patients with Respiratory Symptoms

**DOI:** 10.1371/journal.pone.0051545

**Published:** 2012-12-21

**Authors:** Robert J. Blount, Leah G. Jarlsberg, Kieran R. Daly, William Worodria, J. Lucian Davis, Adithya Cattamanchi, Kpandja Djawe, Alfred Andama, Judith Koch, Peter D. Walzer, Laurence Huang, International HIV-Associated Opportunistic Pneumonias (IHOP) Study

**Affiliations:** 1 Division of Pulmonary and Critical Care Medicine, San Francisco General Hospital, University of California San Francisco, San Francisco, California, United States of America; 2 Division of Infectious Diseases, Department of Internal Medicine, University of Cincinnati, Cincinnati, Ohio, United States of America; 3 Veterans Administration Medical Center, Cincinnati, Ohio, United States of America; 4 Makerere University - University of California San Francisco (MU-UCSF) Research Collaboration, Kampala, Uganda; 5 Department of Medicine, Mulago Hospital, Kampala, Uganda; 6 Division of Epidemiology and Biostatistics, Department of Environmental Health, University of Cincinnati, Cincinnati, Ohio, United States of America; 7 HIV/AIDS Division, San Francisco General Hospital, University of California San Francisco, San Francisco, California, United States of America; Vanderbilt University, United States of America

## Abstract

**Background:**

Little is known about the serologic responses to *Pneumocystis jirovecii* major surface glycoprotein (Msg) antigen in African cohorts, or the IgM responses to Msg in HIV-positive and HIV-negative persons with respiratory symptoms.

**Methods:**

We conducted a prospective study of 550 patients, both HIV-positive (n = 467) and HIV-negative (n = 83), hospitalized with cough ≥2 weeks in Kampala, Uganda, to evaluate the association between HIV status, CD4 cell count, and other clinical predictors and antibody responses to *P. jirovecii*. We utilized ELISA to measure the IgM and IgG serologic responses to three overlapping recombinant fragments that span the *P. jirovecii* major surface glycoprotein: MsgA (amino terminus), MsgB (middle portion) and MsgC1 (carboxyl terminus), and to three variations of MsgC1 (MsgC3, MsgC8 and MsgC9).

**Results:**

HIV-positive patients demonstrated significantly lower IgM antibody responses to MsgC1, MsgC3, MsgC8 and MsgC9 compared to HIV-negative patients. We found the same pattern of low IgM antibody responses to MsgC1, MsgC3, MsgC8 and MsgC9 among HIV-positive patients with a CD4 cell count <200 cells/µl compared to those with a CD4 cell count ≥200 cells/µl. HIV-positive patients on PCP prophylaxis had significantly lower IgM responses to MsgC3 and MsgC9, and lower IgG responses to MsgA, MsgC1, MsgC3, and MsgC8. In contrast, cigarette smoking was associated with increased IgM antibody responses to MsgC1 and MsgC3 but was not associated with IgG responses. We evaluated IgM and IgG as predictors of mortality. Lower IgM responses to MsgC3 and MsgC8 were both associated with increased in-hospital mortality.

**Conclusions:**

HIV infection and degree of immunosuppression are associated with reduced IgM responses to Msg. In addition, low IgM responses to MsgC3 and MsgC8 are associated with increased mortality.

## Introduction

*Pneumocystis jirovecii* continues to be an important cause of pneumonia in immunosuppressed individuals, especially in those with HIV infection who do not have access to or cannot tolerate antiretroviral therapy (ARV) or *Pneumocystis* prophylaxis. Because *Pneumocystis* is not easily cultured, serologic studies have been particularly important in providing insights into *Pneumocystis* exposure, transmission, disease activity and immune responses. For instance, through these studies we have found that healthcare workers with direct patient contact have higher antibody levels to *P. jirovecii* than staff without patient contact, [1] suggesting potential person-to-person transmission. We have also found that the serologic responses to *P. jirovecii* are dependent on geographic location, [2], [3] suggesting geographic variation in the level of *P. jirovecii* exposure, exposure to different strains of *P. jirovecii,* or differences in host immunologic responses to *Pneumocystis* in varied human populations.

While early serologic studies used crude *Pneumocystis* antigen preparations [4]–[10] that often led to poorly reproducible results, recombinant *P. jirovecii* antigens such as major surface glycoprotein (Msg) provide a more standardized and powerful tool for understanding the interaction between the mammalian host and *P. jirovecii*. Major surface glycoprotein represents a family of proteins encoded by multiple genes. [11], [12] Switching of Msg gene expression gives rise to different Msg isoforms resulting in antigenic variation. [13], [14] Msg is also highly immunogenic and contains both B and T cell protective epitopes.[11], [12], [15]–[19] We have developed three overlapping recombinant fragments to the *Pneumocystis jirovecii* major surface glycoprotein: MsgA, MsgB and MsgC1 referring to the amino-terminus, middle portion and carboxyl-terminus of the protein, respectively. [20], [21] Of these three regions, the carboxyl-terminus appears to be most conserved. [22] To better define the reactivity of serum antibodies to this region, we have developed three variations of MsgC1: MsgC3, MsgC8 and MsgC9. [23] In studies using these recombinant Msg fragments, we have found that HIV infection is usually, but not always, associated with decreased serologic responses to *P. jirovecii.* [2], [20], [21], [24] Among HIV-infected persons, we have found that low CD4 cell count, however, has not been associated with reduced serologic responses to Msg, [3], [20], [25] with the exception of one study finding greater serologic responses in patients with active PCP who had CD4 cell counts >50 cells/µl. [26].

Although considerable effort has been devoted to characterizing the IgG responses to recombinant Msg fragments in various populations, little is known about the IgM responses and the factors that influence the magnitude of IgM antibody responses. Furthermore, there is limited serologic data specific to sub-Saharan African cohorts, where the majority of HIV-positive persons reside. Therefore, we conducted a 14-month prospective study of hospitalized patients with cough ≥2 weeks and suspected pneumonia in Kampala, Uganda as part of the International HIV-associated Opportunistic Pneumonias (IHOP) Study. Our objectives were to evaluate the effects of HIV status, CD4 cell count, and other clinical predictors on IgM and IgG antibody responses to *P. jirovecii* recombinant Msg fragments, and to evaluate antibody responses as predictors for mortality.

## Methods

### Study Population

Between May 2007 and June 2008, we screened consecutive adults (>18 years old) admitted to Mulago Hospital in Kampala, Uganda. Those with cough ≥2 weeks but <6 months in duration were eligible for the study. Those on anti-TB therapy or with evidence of heart failure at the time of screening were excluded.

### Data Collection

After obtaining written, informed consent, we collected demographic and clinical information by a standardized questionnaire. We obtained a cigarette smoking history and determined total pack-years of smoking. We calculated “cooking smoke-years,” hours of cooking over wood and/or charcoal each day multiplied by years of cooking with these biomass fuels, to estimate indoor biomass smoke exposure. We defined rainy season months (March, April, May and November) as those months in which the average rainfall was >120 mm as reported at www.bbc.co.uk/weather/. [27] Among HIV-infected patients, we obtained information on use of antiretroviral therapy and PCP prophylaxis.

### Diagnostic Evaluation

We tested all participants for HIV who did not already carry this diagnosis, using a previously described sequential testing algorithm incorporating three rapid enzyme immunoassays. [28] We tested each finger-stick blood specimen using two rapid enzyme immunoassays: Determine (Abbott Laboratories, Abbott Park, IL, USA) and Uni-Gold (Trinity Biotech, Wicklow, Ireland). If test results on the specimen were concordant, we performed no further HIV testing. However, if test results were discordant, we performed a third rapid enzyme immunoassay using Stat-Pak (Chembio, Medford, NY, USA). Those with 2 out of 3 tests positive were diagnosed with HIV, and those with 2 out of 3 tests negative were considered to be HIV-negative. We determined CD4 cell counts on all HIV-infected participants. We evaluated all participants for tuberculosis and other HIV-associated pulmonary diseases by a previously described protocol.[29]–[31] In brief, each participant submitted two sputum specimens for acid-fast bacilli (AFB) examination by Ziehl Neelsen method and for mycobacterial culture on Lowenstein-Jensen media. We performed bronchoscopy with bronchoalveolar lavage (BAL) on consenting AFB smear-negative patients if referred by the ward attending. We performed bronchoscopic examination for endobronchial Kaposi sarcoma. We analyzed BAL fluid for *Mycobacterium tuberculosis* using AFB smear and culture, *Pneumocystis jirovecii* using a modified Giemsa stain, and other fungi using potassium hydroxide smear, India ink stain and culture on Sabouraud agar. At the University of North Carolina we determined *Pneumocystis* colonization status by performing PCR on BAL specimens, targeting *P. jirovecii* mitochondrial large subunit rRNA. [32] Colonization was defined as BAL fluid positive for *P. jirovecii* by PCR but negative by Giemsa staining. Participants were followed throughout their hospitalization. Serum was collected from each patient at the time of enrollment, stored at −20°C and shipped to University of Cincinnati and Cincinnati Veterans Administration Medical Center, where it was stored at −80°C prior to analysis.

### Recombinant Antigens

We used PCR with AmpliTaq enzyme (Applied Biosystems, Carlsbad, CA, USA) to amplify three overlapping sections spanning the entire length of Msg gene: Msg_15-1119_, Msg_729-2282_, and Msg_2015-3332_ (subscripts denote number of nucleotides). [20] We used genomic DNA from *P. jirovecii* infected human lung as the template for Msg_15-1119_, and a λgt11 clone of human derived Msg gene as the template for Msg_729-2282_ and Msg_2015-3332_. We then inserted the amplified gene segments into the pET30 *E. coli* expression system (Novagen, Madison, WI, USA) for generation of MsgA, MsgB, and MsgC1 recombinant proteins encoded by the gene segments Msg_15-1119_, Msg_729-2282_, and Msg_2015-3332_, respectively. We then purified these proteins by affinity chromatography. Using a similar process and *P. jirovecii* infected human lung, we generated MsgC3, MsgC8, and MsgC9 recombinant proteins from the same gene region as for MsgC1. [23] The overlapping recombinant fragments generated are representative of the entire major surface glycoprotein: MsgA (amino-terminus), MsgB (middle portion) and MsgC fragments (carboxyl-terminus).

### IgM and IgG ELISA

Using previously developed ELISA protocols for IgG [3], [20], [21], [23], [25], [26] and IgM, [25] we measured serologic responses to each recombinant Msg fragment. We tested serum specimens and standard reference sera against the recombinant Msg fragments, using phosphate-buffered saline (PBS) without Msg as the negative control. We corrected the reactivity of each serum specimen to Msg by subtracting the reactivity of each serum specimen to PBS (mean optical density (OD) with Msg – mean OD with PBS alone) and quantified the results using methods described by Bishop and Kovacs. [33] We prepared a standard serum with specificity for each Msg construct by mixing the sera from 4 to 6 specimens with high reactivity for the specific construct. We selected these specimens by testing banks of sera from blood donors and HIV-infected patients. The initial standard pool for each antigen was defined as having a value of 100 U in 100 ul of a 1∶100 dilution. We used the same standard pools throughout the study, and as a further measure to ensure consistency between assays, we titrated subsequent standard pools against those initial standards. From the standard pool, we generated a standard curve for each Msg construct on each day the assay was used. We used this curve to calculate the units of reactivity to the Msg construct. We diluted test serum samples at 1∶100 to 1∶200 to fit the linear portion of the standard curves. Taking into account the dilution, we then calculated units of reactivity.

### Ethics Approval

The study was approved by the institutional review boards at University of California San Francisco, University of Cincinnati, Mulago Hospital, Makerere University, and the Uganda National Council for Science and Technology.

### Statistical Analysis

We evaluated associations between clinical predictors and antibody responses to Msg fragments using tobit regression for left and right censored data, with log-transformed Msg antibody level as the dependent variable. Mean antibody levels were exponentiated and presented as tobit-estimated geometric means with 95% confidence intervals (CI). Predictors tested were HIV status, age, sex, cigarette pack-years, cooking smoke-years, and specimen collection during rainy season. In HIV-positive individuals additional factors tested were: CD4 cell count; antiretroviral (ARV) use; *Pneumocystis* pneumonia (PCP) prophylaxis; *P. jirovecii* colonization; and diagnosis of active PCP, active tuberculosis (TB), and other symptomatic pulmonary infections. If predictors were associated with Msg antibody levels at p<0.2 in bivariate analysis, we adjusted for them in multivariate models. We used logistic regression to model in-hospital death as the outcome with each logged Msg level as the predictor, controlling for age, CD4 cell count, ARV use, PCP prophylaxis, and PCP diagnosis. Odds of death were calculated per 5 original Msg units of increase (equivalent to 1.61 logged Msg units).

## Results

### Cohort

**Figure 1:**
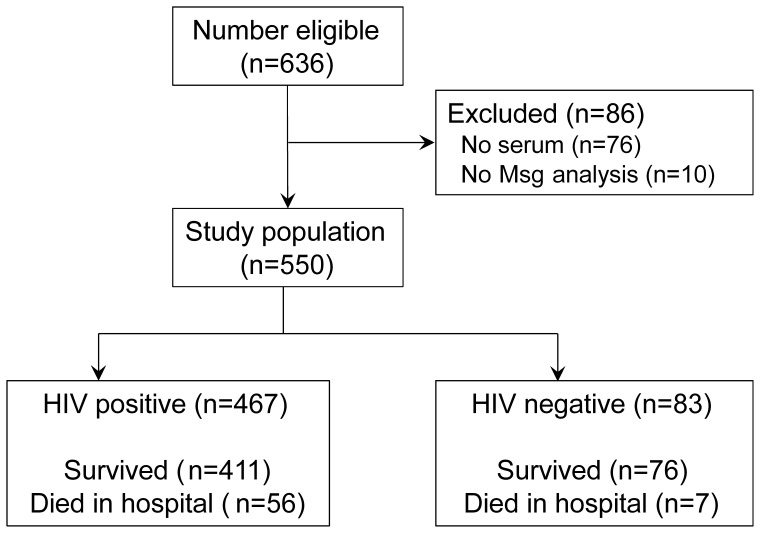
Study enrollment flow diagram.

Of 636 patients eligible for the study, 550 were enrolled (Figure 1). Four hundred sixty-seven (85%) were HIV-positive and 83 (15%) were HIV-negative (Table 1). HIV-positive participants were significantly younger than those without HIV (mean age 34.2 years vs. 43.7 years, p<0.001), and a higher percentage of HIV-positive individuals were female (54.8% vs. 32.5%, p<0.001). The proportion of cigarette smokers (ever and current) and the proportion who cooked meals with wood and/or charcoal stoves indoors was similar between HIV-positive and HIV-negative patients. However, HIV-positive patients had smoked fewer pack-years (median 3.0 vs. 9.0, p = 0.002) and reported less cumulative exposure to indoor biomass smoke as measured by cooking smoke-years (median 57.7 vs. 79.6, p = 0.01).

**Table 1:** Demographics for all-comers and by HIV status.

Patient characteristics	All	HIV Positive	HIV Negative	p value
N	550	467	83	
Mean age ±SD	35.7±10.8	34.2±8.8	43.7±16.1	<0.001
Age >50 years	53 (9.6%)	25 (5.4%)	28 (33.7%)	<0.001
Female sex	283 (51.5%)	256 (54.8%)	27 (32.5%)	<0.001
Current smoker	29 (5.3%)	23 (4.9%)	6 (7.2%)	0.42
Ever smoker	162 (29.5%)	133 (28.5%)	29 (34.9%)	0.23
Median pack years smoking (IQR) (N = 161)	3.0 (0.8–10.0)	3.0 (0.6–9.8)	9.0 (2.0–20.0)	0.002
Currently use wood/charcoal stove	399 (72.6%)	345 (73.9%)	54 (65.1%)	0.10
Mean cooking smoke-years ±SD (N = 388)	60.7±45.2	57.7±43.1	79.6±53.2	0.01
Specimen collected during the rainy season	363 (66.0%)	304 (65.1%)	59 (71.1%)	0.29
Final diagnosis:				
Pulmonary tuberculosis	303 (55.1%)	267 (57.2%)	36 (43.4%)	Referent
* Pneumocystis* pneumonia	6 (1.1%)	6 (1.3%)	0 (0%)	0.56*
Other	102 (18.6%)	72 (15.4%)	30 (36.1%)	0.02
Unknown	139 (25.3%)	122 (26.1%)	17 (20.5%)	0.75
Death in hospital	63 (11.5%)	56 (12.0%)	7 (8.4%)	0.35
**Characteristics associated with HIV**				
Newly diagnosed HIV infection		140 (30.0%)		
ARV at enrollment		71 (15.2%)		
On PCP prophylaxis (N = 466)		256 (54.9%)		
Median CD4 count (IQR) (N = 463)		50 (12–160)		
CD4 count <200 (N = 463)		371 (80.1%)		
CD4 count <50 (N = 463)		231 (49.9%)		

SD indicates standard deviation; IQR, interquartile range; ARV, antiretroviral therapy; PCP, *Pneumocystis* pneumonia.

* A value of 0.5 was substituted for the zero cell.

Among HIV-positive participants, 30.0% were newly diagnosed with HIV infection at the time of enrollment (Table 1). Two hundred fifty-six (54.9%) of all HIV-positive participants were taking PCP prophylaxis at the time of enrollment: 253 (99.0%) on trimethoprim/sulfamethoxazole and 3 on dapsone. Only 71 (15.2%) were on ARV therapy at the time of enrollment. Of these, the specific ARVs taken were known for 56. The most common ARV combination taken was stavudine, lamivudine and nevirapine, n = 21. Most HIV-positive patients were severely immunosuppressed; 80.1% had a CD4 cell count <200 cells/µl and 49.9% had a CD4 count <50 cells/µl at enrollment. In-hospital mortality was high: all participants, 11.5%; HIV-positive participants, 12.0%; and HIV-negative participants, 8.4%. There was no statistically significant difference in mortality between the HIV-positive and HIV-negative groups (p = 0.35).

There were no statistically significant differences between those excluded (n = 86) from the study and those enrolled with two exceptions: those excluded were more likely to have an undetermined final diagnosis compared to those included (48.2% vs. 25.3%, p<0.001), and had less cooking smoke-years exposure (20.0 vs. 60.7, p<0.001).

### Predictors of Antibody Responses to *P. jirovecii* Msg

#### HIV infection

HIV-positive patients had significantly lower IgM antibody levels to most recombinant Msg fragments compared with HIV-negative participants, even after adjustment for age, sex, cigarette pack-years smoked, cooking smoke-years, and season of hospitalization/specimen collection (Table 2). The geometric mean IgM antibody levels to all Msg carboxyl-terminus fragments (MsgC1, MsgC3, MsgC8 and MsgC9) were significantly decreased in HIV-positive patients compared to HIV-negative patients. IgM antibody levels to these Msg fragments were 4.4-fold to 9.5-fold lower in the HIV-positive cohort compared with the HIV-negative cohort: MsgC1, 4.0 vs. 19.7 (p<0.001); MsgC3, 1.1 vs. 10.5 (p<0.001); MsgC8, 1.5 vs. 9.7 (p<0.001); and MsgC9, 3.0 vs. 13.1 (p<0.001). In contrast, geometric mean IgM antibody levels to MsgA and MsgB, although decreased in unadjusted models, were not significantly decreased when adjusted for covariates. While IgM levels to most Msg fragments were decreased, IgG levels were only decreased significantly (p = 0.01) to MsgA in HIV-positive patients compared with HIV-negative patients. Of the 467 HIV-positive patients enrolled in the study, 140 (30.0%) were newly diagnosed with HIV. In comparison to those with an established HIV diagnosis, newly diagnosed patients appeared to have more robust IgM and IgG serologic responses (1.3–3.3 fold higher antibody levels; p values <0.001–0.04) to all Msg constructs tested except for IgG to MsgA and MsgB. However, these differences were not statistically significant in multivariate analysis.

**Table 2:** Estimated geometric mean IgM and IgG antibody levels to Msg fragments by HIV status.

	HIV-Positive	HIV-Negative		
	EGM Antibody level, (95%CI)	EGM Antibody level, (95%CI)	Unadjusted Beta (SE), p value	Adjusted* Beta (SE), p value
N	467	83		
**IgM**				
MsgA	17.9 (15.6–20.4)	33.3 (23.8–46.6)	–0.62 (0.18), <0.001	–0.36 (0.19), 0.05
MsgB	3.8 (3.0–4.6)	7.3 (4.3–12.6)	–0.72 (0.27), 0.01	–0.38 (0.29), 0.19
MsgC1	4.0 (3.1–5.0)	19.7 (12.5–31.2)	–1.57 (0.29), <0.001	–1.16 (0.31), <0.001
MsgC3	1.1 (0.9–1.5)	10.5 (6.5–16.8)	–2.17 (0.30), <0.001	–1.91 (0.31), <0.001
MsgC8	1.5 (1.2–2.0)	9.7 (6.1–15.4)	–1.79 (0.29), <0.001	–1.46 (0.31), <0.001
MsgC9	3.0 (2.5–3.6)	13.1 (8.6–19.8)	–1.46 (0.23), <0.001	–1.35 (0.25), <0.001
**IgG**				
MsgA	1.1 (0.7–1.6)	7.4 (4.2–13.2)	–1.62 (0.43), <0.001	–1.18 (0.45), 0.01
MsgB	0.12 (0.07–0.23)	0.21 (0.06–0.78)	–0.58 (0.52), 0.26	–0.58 (0.52), 0.26
MsgC1	5.5 (4.4–7.0)	9.8 (6.0–15.9)	–0.52 (0.29), 0.08	–0.25 (0.31), 0.43
MsgC3	62.6 (55.6–70.4)	81.0 (64.1–102)	–0.26 (0.15), 0.09	–0.18 (0.16), 0.26
MsgC8	45.6 (40.7–50.9)	52.1 (41.8–64.8)	–0.13 (0.14), 0.36	–0.07 (0.15), 0.65
MsgC9	24.7 (21.3–28.6)	38.6 (28.9–51.5)	–0.44 (0.19), 0.02	–0.29 (0.20), 0.16

EGM indicates estimated geometric mean; CI, confidence interval; SE, standard error; Msg, *Pneumocystis jirovecii* major surface glycoprotein.

* Adjusted for any characteristics (age >50, sex, cigarette pack years smoked, years of wood/charcoal cooking smoke exposure, and specimen collection during the rainy season) that were associated (p<0.2) with Msg antibody levels in unadjusted analysis.

#### CD4 cell count

As with HIV infection, the severity of immunosuppression quantified by CD4 cell count was also associated with significant decreases in IgM antibody levels to all *P. jirovecii* Msg carboxyl-terminus fragments (Table 3). Among HIV-positive patients, those with a CD4 cell count <200 cells/µl had significantly lower geometric mean IgM antibody levels to MsgC1, MsgC3, MsgC8 and MsgC9 compared to those with a CD4 cell count ≥200 cells/µl, both in unadjusted analysis and when adjusted for confounding variables. Geometric mean IgM antibody levels in HIV-positive participants with CD4 count <200 cells/µl were 1.8-fold to 2.5-fold lower than seen in those HIV-positive participants with CD4 count ≥200 cells/µl: MsgC1, 3.6 vs. 6.6 (p = 0.02); MsgC3, 1.0 vs. 2.1 (p = 0.03); MsgC8, 1.3 vs. 3.3 (p = 0.01); and MsgC9, 2.7 vs. 5.2 (p = 0.01). Although CD4 cell count was associated with IgM responses, it was not predictive of IgG responses, except for IgG to MsgC1 (6.2 vs. 3.2, p = 0.047).

**Table 3:** Mean Msg antibody levels by CD4 count in HIV-positive patients.

	CD4 count <200	CD4 count ≥200		
	EGM Antibody level, (95%CI)	EGM Antibody level, (95%CI)	Unadjusted Beta (SE), p value	Adjusted* Beta (SE), p value
N	371	92		
**IgM**				
MsgA	16.8 (14.5–19.5)	23.3 (17.2–31.5)	–0.33 (0.17), 0.06	–0.26 (0.17), 0.13
MsgB	3.4 (2.7–4.3)	5.8 (3.7–9.2)	–0.53 (0.26), 0.04	–0.47 (0.26), 0.07
MsgC1	3.6 (2.8–4.6)	6.6 (3.5–12.4)	–0.74 (0.29), 0.01	–0.65 (0.29), 0.02
MsgC3	1.0 (0.8–1.4)	2.1 (1.1–3.7)	–0.78 (0.30), 0.01	–0.63 (0.30), 0.03
MsgC8	1.3 (1.0–1.7)	3.3 (2.0–5.4)	–0.87 (0.29), 0.003	–0.80 (0.29), 0.01
MsgC9	2.7 (2.2–3.3)	5.2 (3.5–7.8)	–0.67 (0.23), 0.003	–0.56 (0.23), 0.01
**IgG**				
MsgA	1.0 (0.6–1.6)	1.3 (0.5–3.4)	–0.33 (0.47), 0.48	–0.22 (0.47), 0.64
MsgB	0.11 (0.05–0.22)	0.25 (0.09–0.72)	–0.30 (0.51), 0.56	–0.30 (0.51), 0.56
MsgC1	6.2 (4.8–8.0)	3.2 (1.7–6.0)	0.53 (0.30), 0.08	0.59 (0.30), 0.047
MsgC3	65.2 (57.3–74.1)	54.9 (41.0–73.3)	0.17 (0.15), 0.27	0.22 (0.15), 0.14
MsgC8	47.0 (41.5–53.3)	40.2 (31.1–51.9)	0.16 (0.14), 0.27	0.19 (0.14), 0.18
MsgC9	25.4 (21.4–30.0)	22.5 (16.3–31.2)	0.12 (0.19), 0.53	0.17 (0.19), 0.37

EGM indicates estimated geometric mean; CI, confidence interval; SE, standard error; Msg, *Pneumocystis jirovecii* major surface glycoprotein.

* Adjusted for any characteristics (age >50, sex, on ARV at admission, received PCP prophylaxis, diagnosed with PCP, cigarette pack-years smoked, years of wood/charcoal cooking smoke exposure, and specimen collection during the rainy season) that were associated (p<0.2) with Msg antibody levels in unadjusted analysis.

#### PCP and *P. jirovecii* colonization

Of 467 HIV-infected patients enrolled in the study, 142 AFB smear negative patients were eligible and referred by the ward attending for bronchoscopy. One hundred twenty-two were consented, cleared by pre-bronchoscopy history and physical, and underwent bronchoscopy with BAL evaluation for *Pneumocystis* infection and colonization. Of those who underwent bronchoscopy, 6 were diagnosed with PCP by modified Giemsa staining, 7 additional patients were colonized (Giemsa negative but PCR positive), and 109 had no evidence of PCP infection or *Pneumocystis* colonization. Patients with active PCP demonstrated higher IgM and IgG antibody responses to all the Msg fragments when compared with *P. jirovecii* colonization. However, this was only statistically significant in multivariate analysis for IgM to MsgA (42.6 vs. 15.6, p = 0.002); IgM to MsgB (19.8 vs. 2.0, p<0.001); and IgG to MsgC1 (43.6 vs. 11.0, p = 0.02). Antibody levels to the Msg fragments were similar between PCP negative and *P. jirovecii* colonized groups, with no significant differences.

#### Pulmonary tuberculosis

Of the 550 enrolled in-patients, 303 (55.1%) were diagnosed with pulmonary tuberculosis. There were no statistically significant associations between the diagnosis of active pulmonary TB and serologic responses to the different Msg fragments.

#### Other predictors

HIV-positive patients taking PCP prophylaxis at the time of enrollment had decreased antibody levels to Msg fragments when compared with HIV-positive patients not on PCP prophylaxis (Table 4). Those on PCP prophylaxis had significantly lower geometric mean IgM levels to MsgC3 and MsgC9, and significantly lower geometric mean IgG levels to MsgA, MsgC1, MsgC3 and MsgC8. In contrast, in HIV-positive patients, amount of cigarette smoking as indicated by cigarette pack-years was associated with significantly increased IgM antibody responses to MsgC1 and MsgC3, but was not associated with IgG responses to any of the Msg fragments (data not shown). The other predictors that we evaluated (age, sex, ARV use, cook smoke exposure, and rainy season) were not associated with antibody responses to Msg in either unadjusted or adjusted analyses.

**Table 4:** Mean Msg antibody levels by PCP prophylaxis in the HIV-positive patients.

	PCP prophylaxis	No prophylaxis		
	EGM Antibody level, (95%CI)	EGM Antibody level, (95%CI)	Unadjusted Beta (SE), p value	Adjusted* Beta (SE), p value
**N**	256	210		
**IgM**				
MsgA	14.8 (12.5–17.6)	22.9 (18.8–28.0)	–0.44 (0.13), 0.001	–0.26 (0.14), 0.07
MsgB	3.1 (2.3–4.1)	4.8 (3.5–6.5)	–0.44 (0.21), 0.03	–0.22 (0.22), 0.33
MsgC1	3.2 (2.4–4.4)	5.2 (3.6–7.5)	–0.55 (0.23), 0.02	–0.23 (0.25), 0.36
MsgC3	0.6 (0.4–1.0)	2.1 (1.5–3.0)	–1.08 (0.25), <0.001	–0.82 (0.26), 0.001
MsgC8	1.1 (0.8–1.6)	2.3 (1.6–3.3)	–0.74 (0.24), 0.002	–0.44 (0.25), 0.08
MsgC9	2.2 (1.7–2.9)	4.4 (3.4–5.7)	–0.68 (0.18), <0.001	–0.55 (0.20), 0.004
**IgG**				
MsgA	0.7 (0.4–1.3)	1.7 (0.9–3.0)	–0.74 (0.38), 0.048	–0.77 (0.38), 0.04
MsgB	0.09 (0.04–0.23)	0.17 (0.07–0.40)	–0.58 (0.42), 0.16	–0.52 (0.42), 0.22
MsgC1	4.2 (3.0–6.0)	7.7 (5.6–10.6)	–0.52 (0.24), 0.03	–0.55 (0.25), 0.03
MsgC3	53.6 (44.9–64.1)	75.8 (65.5–87.6)	–0.34 (0.12), 0.004	–0.26 (0.13), 0.04
MsgC8	40.6 (34.5–47.9)	52.5 (45.3–60.9)	–0.25 (0.11), 0.03	–0.25 (0.11), 0.03
MsgC9	21.4 (17.4–26.3)	29.7 (24.1–36.6)	–0.32 (0.15), 0.04	–0.29 (0.15), 0.06

EGM indicates estimated geometric mean; CI, confidence interval; SE, standard error; Msg, *Pneumocystis jirovecii* major surface glycoprotein.

* Adjusted for any characteristics (age >50, sex, on ARV at admission, CD4 count <200, diagnosed with PCP, cigarette pack-years smoked, years of wood/charcoal cooking smoke exposure, and specimen collection during the rainy season) that were associated (p<0.2) with Msg antibody levels in unadjusted analysis.

#### Predictors of mortality

Of all the fragments tested, only IgM antibody levels to MsgC3 and MsgC8 were associated with in-hospital mortality (Table 5). For every 5 unit increase in IgM levels to MsgC3 and MsgC8, HIV-positive participants had odds of 0.60 and 0.63 for in-hospital mortality (OR 0.60, 95%CI 0.39–0.93 and OR 0.63, 95%CI 0.43–0.94) respectively. These associations remained statistically significant when adjusting for potential confounders (age, CD4 cell count, ARV use, PCP prophylaxis and PCP diagnosis). Age, CD4 cell count, ARV use, PCP prophylaxis and PCP diagnosis were evaluated as independent predictors of mortality (Table 5). Both CD4 count and PCP diagnosis were found to be predictive of mortality in multivariate analysis. For every 50 cell/µl increase in CD4 cell count, HIV-positive participants had 0.79 times the odds of in-hospital mortality (OR 0.79, 95%CI 0.67–0.94). HIV-positive participants with the diagnosis of PCP had 5.74 times the odds of in-hospital mortality (OR 5.74, 95%CI 1.09–30.1), compared to those not diagnosed with PCP.

**Table 5:** Odds of hospital mortality by predictors tested in HIV-positive participants.†

	Unadjusted	Adjusted*
	Odds Ratio (95%CI), p value	Odds Ratio (95%CI), p value
Age	0.99 (0.72–1.36), 0.95	0.94 (0.67–1.31), 0.71
CD4 count	0.79 (0.67–0.94), 0.01	0.79 (0.67–0.94), 0.01
ARV at diagnosis	1.63 (0.81–3.26), 0.17	2.18 (0.99–4.84), 0.05
PCP prophylaxis	0.94 (0.54–1.65), 0.83	0.68 (0.36–1.31), 0.25
PCP diagnosis	7.70 (1.52–39.1), 0.01	5.74 (1.09–30.1), 0.04
**IgM**		
MsgA	0.89 (0.64–1.23), 0.46	0.89 (0.63–1.26), 0.52
MsgB	0.87 (0.65–1.18), 0.37	0.86 (0.62–1.18), 0.34
MsgC1	0.90 (0.68–1.17), 0.42	0.94 (0.71–1.26), 0.69
MsgC3	0.60 (0.40–0.90), 0.01	0.60 (0.39–0.93), 0.02
MsgC8	0.62 (0.43–0.90), 0.01	0.63 (0.43–0.94), 0.02
MsgC9	0.78 (0.55–1.10), 0.16	0.80 (0.55–1.15), 0.22
**IgG**		
MsgA	0.86 (0.67–1.10), 0.23	0.88 (0.67–1.14), 0.32
MsgB	0.89 (0.60–1.32), 0.56	0.88 (0.58–1.32), 0.52
MsgC1	0.93 (0.72–1.20), 0.56	0.91 (0.69–1.19), 0.49
MsgC3	0.97 (0.68–1.38), 0.86	1.03 (0.70–1.51), 0.89
MsgC8	0.94 (0.65–1.36), 0.74	0.90 (0.61–1.33), 0.59
MsgC9	1.02 (0.75–1.37), 0.92	1.03 (0.75–1.43), 0.84

† Odds of mortality are reported per 5 unit increase in antibody levels to Msg, 10 year increase in age, and 50 cells/µl increase in CD4 count.

* Adjusted for the following characteristics: age, CD4 count, on ARV at admission, PCP prophylaxis, and diagnosed with PCP.

## Discussion

In this prospective study, we evaluated the association between HIV status, CD4 cell count and a number of other clinical predictors on IgM and IgG antibody responses to *P. jirovecii* Msg fragments in a Ugandan cohort of HIV-positive and HIV-negative participants with cough ≥2 weeks. We found that those who were HIV-positive and those with HIV and a CD4 cell count <200 cells/µl had significantly decreased IgM responses to *Pneumocystis* Msg fragments, and that other clinical predictors such as PCP prophylaxis and smoking were also associated with antibody responses to Msg. Additionally, we found that low IgM responses to certain Msg fragments were associated with increased in-hospital mortality.

In our study, HIV status and CD4 cell count were both associated with IgM antibody responses to *P. jirovecii* Msg. It is unclear why IgM serologic responses were diminished in those with HIV and with more severe immunodeficiency, while IgG serologic responses were not diminished. There have been few studies evaluating IgM serologic responses to *P. jirovecii* in HIV-positive compared to HIV-negative individuals. Of these, the majority have found low or absent IgM responses to *Pneumocystis* antigen in HIV-positive individuals [7], [8], [34], [35], consistent with our results. The diminished IgM responses seen in those with HIV and more severe immunosuppression could very well reflect typical immunoglobulin class switching from IgM predominant B cell responses to IgG predominant responses. Those with HIV and greater immunosuppression could have increased *P. jirovecii* exposure and organism burden resulting in heightened stimulation of the humoral immune system and more advanced IgM to IgG class switching compared with HIV-negative participants and those with less severe immunosuppression. Alternatively, the decreased humoral responses seen could be related to HIV immunosuppression itself. In addition to its detrimental impact on CD4 cell count, HIV infection plays a significant role in humoral immune dysfunction. Although polyclonal B cell activation and hypergammaglobulinemia are often seen in HIV-infected persons, [36]–[42] the humoral responses to many specific antigens have been found to be decreased. HIV-positive individuals have decreased specific antibody responses to a wide host of pathogens such as malaria parasites [43], [44], measles virus [37], [45] *Campylobacter jejuni* [46], *Giardia lamblia* [47], *Salmonella spp.*, *Streptococcus pneumoniae* [45], [48]–[51] and *Haemophilus influenzae*. [52] This explanation does not take into account, however, the selective decrease in IgM responses and not IgG responses. Moir et al. have described several mechanisms of B-cell dysfunction in HIV-infected persons [53] that could contribute to our findings, including expansion of dysfunctional B cell subpopulations such as exhausted B cells, [54] increased immune-cell turnover with net loss of B cells, [55] and decreased memory B cell responses [37] with the specific loss of IgM memory B cells. [56] Although the prevalence of PCP and *P. jirovecii* colonization was low in this Ugandan cohort, antibody responses were quite common. This has also been seen in HIV-positive patients and healthy controls without PCP in prior studies evaluating serologic responses to Msg.[1]–[3], [20], [21], [23], [25], [26], [57] The prevalence of IgG seropositivity is likely due to significant exposure to *Pneumocystis jirovecii* in the general population, with infection often first occurring at a young age. Indeed, 85% of infants in one study were seropositive by the age of 20 months, many of them converting during respiratory illnesses thought to be reflective of mild *Pneumocystis* infections. [57], [58] IgM seropositivity, on the other hand, possibly represents new exposure to different strains of *P. jirovecii*. Significant levels of IgG and IgM responses to Msg fragments despite a low prevalence of *P. jirovecii* colonization suggest that exposure to *Pneumocystis* is relatively common but that the majority of those exposed do not become persistently colonized.

Low antibody levels were associated with increased in-hospital mortality in this study, again seen specifically with IgM and not with IgG. These findings remained significant even after controlling for CD4 cell count, suggesting a humoral immune dysfunction, independent of cellular immunosuppression, impacting mortality. As mentioned above, HIV-positive individuals have been found to have specific IgM memory B cell dysfunction. [53], [56] These findings should be interpreted with caution, however, as IgM responses to only two of the MsgC fragments tested were significantly associated with mortality, increasing the likelihood that the findings could be due to chance alone, or influenced by confounding or effect modification from unmeasured variables. As expected, both CD4 cell count and PCP diagnosis were also associated with mortality.

In this and prior studies, results have been most significant for the MsgC fragments, compared to MsgA and MsgB fragments. MsgC fragments, for instance, have been particularly useful in distinguishing individuals with active or past PCP from those with no history of PCP. [3], [20], [25], [26] In contrast, we have found less consistent serologic responses to the MsgA and MsgB fragments. [1], [21], [26] Multiple genes encode the major surface glycoprotein giving rise to variability in protein expression. [11], [12] This variability appears to be most pronounced in the amino-terminus and middle portion of the protein, while the carboxyl-terminus region, represented by the MsgC fragments in our model, appears to be the most antigenically conserved region. [22].

This property likely contributes to the immunologic, epidemiologic, and clinical utility of the MsgC fragments.

Our study is limited by the low prevalence of both PCP and *P. jirovecii* colonization which has significantly impacted our ability to detect associations between these important predictors and serologic responses to Msg fragments in Uganda. These results are not surprising, as several other studies have also found low PCP prevalences among those with HIV in East Africa.[59]–[62] Despite this limitation, we were able to adequately evaluate several other predictors of antibody responses to Msg given the large number of participants in the study.

In conclusion, this is one of the first large studies to evaluate IgM antibody responses, in addition to IgG, to *P. jirovecii* recombinant Msg antigens. We have found that HIV infection and degree of immunosuppression are associated with reduced IgM responses to multiple Msg fragments, suggesting increased immunoglobulin class switching or a specific humoral immunodeficiency to *P. jirovecii* in HIV-positive individuals. Low IgM levels to MsgC3 and MsgC8 were also associated with increased in-hospital mortality. Our results add important insight on the humoral responses to *P. jirovecii* in those with HIV in Uganda.
